# First prosecution of a Dutch doctor since the Euthanasia Act of 2002: what does the verdict mean?

**DOI:** 10.1136/medethics-2019-105877

**Published:** 2019-12-05

**Authors:** Eva Constance Alida Asscher, Suzanne van de Vathorst

**Affiliations:** 1 Medical Ethics/General Practice, Amsterdam UMC–Locatie AMC, Amsterdam, North Holland, Netherlands; 2 Medical Ethics and Philosophy of Medicine, Erasmus Medical Centre, Rotterdam, Netherlands

**Keywords:** Euthanasia (Active voluntary), Advance Directives, Dementia, Legal Case

## Abstract

On 11 September 2019, the verdict was read in the first prosecution of a doctor for euthanasia since the Termination of Life on Request and Assisted Suicide (Review Procedures) Act of 2002 was installed in the Netherlands. The case concerned euthanasia on the basis of an advance euthanasia directive (AED) for a patient with severe dementia. In this paper we describe the review process for euthanasia cases in the Netherlands. Then we describe the case in detail, the judgement of the Regional Review Committees for Termination of Life on Request and Euthanasia (RTE) and the judgement of the medical disciplinary court. Both the review committees and the disciplinary court came to the conclusion there were concerns with this case, which mainly hinged on the wording of the AED. They also addressed the lack of communication with the patient, the absence of oral confirmation of the wish to die and the fact that the euthanasia was performed without the patient being aware of this. However, the doctor was acquitted by the criminal court as the court found she had in fact met all due care criteria laid down in the act. We then describe what this judgement means for euthanasia in the Netherlands. It clarifies the power and reach of AEDs, it allows taking conversations with physicians and the testimony of the family into account when interpreting the AED. However, as a practical consequence the prosecution of this physician has led to fear among doctors about prosecution after euthanasia.

## Introduction

Recently the first prosecution of a Dutch physician since the 2002 Termination of Life on Request and Assisted Suicide (Review Procedures) Act ended in acquittal. The verdict sheds some light on the requirements for an advance euthanasia directive (AED) in the Netherlands. Moreover, there are practical consequences of the prosecution, which may not be in line with the verdict. Here we describe the process of review, this case and the verdict (also of earlier judgements) to conclude what this case means for the Dutch euthanasia practice and AEDs.

## The framework for review of euthanasia cases

The Termination of Life on Request and Assisted Suicide (Review Procedures) Act stipulates an exception from the prohibition to aid in suicide or to kill in the penal code, but only for physicians if they report the euthanasia or assisted suicide and act according to the requirements of due care as stipulated in the Act.^1^ These requirements are that the physician must:

- Be satisfied that the patient’s request is voluntary and well considered.
- Be satisfied that the patient’s suffering is unbearable, with no prospect of improvement.
- Have informed the patient about his situation and his prognosis.
- Have come to the conclusion, together with the patient, that there is no reasonable alternative in the patient’s situation.
- Have consulted at least one other, independent physician, who must see the patient and give a written opinion on whether the due care criteria set out in (a) to (d) have been fulfilled.
- Have exercised due medical care and attention in terminating the patient’s life or assisting in his suicide.

A physician who has performed euthanasia must then notify the municipal pathologist, who completes a form after the postmortem examination. The physician also has to provide the pathologist with a detailed report and the independent physician’s report.

The Act stipulates in section 2.2 that a patient aged 16 or over who is decisionally competent may draw up an advance directive, setting out a request for euthanasia. If at some point the patient is no longer capable of expressing his will, the physician may accept the advance directive as a request pursuant to section 2 (1)(a) of the Act.^1 2^ The advance directive thus has the same status as an oral request for euthanasia.

The Act further establishes the Regional Review Committees for Termination of Life on Request and Assisted Suicide (RTE).^1^ They have to judge whether the euthanasia was performed with due care. The RTEs comprised a lawyer (also the chairperson), a physician and a philosophical member (usually an ethicist or theologian). The RTEs review all (reported) cases of euthanasia and assisted suicide. Their experience is bundled in the Euthanasia Code 2018, which gives guidelines on how to interpret the due care criteria.^2^ If the RTEs find the criteria for due care were met, they notify the physician and there is no further follow-up (this is the case for the vast majority of the cases: 6120 out of 6126 cases in 2018).^3^ If they conclude the criteria are not met (six cases in 2018), the cases are forwarded to Health Care Inspectorate and the Public Prosecution for further investigation. Both can do an independent investigation as their focus is different. The Health Care Inspectorate investigates whether the physician acted according to professional standards, whereas the Prosecution focuses on whether there is a case for criminal liability. Since the instalment of the Act (which is largely based on the case law from before) in 2002, physicians have faced disciplinary court on a number of occasions (both as a result of the investigation of the Inspectorate and as a result of complaints) as happened in this case, but it was the first case also brought before criminal court.

## The case

The case concerned a patient with severe dementia who had written an AED when she was still competent (see also Miller *et al* 2019).^4^ In October 2012 the patient was diagnosed with Alzheimer’s disease and shortly thereafter she signed a written euthanasia request (the AED) complete with a dementia clause (an additional personal declaration about the wishes regarding euthanasia in the case of dementia). The patient discussed the AED with her general practitioner (GP) and treating gerontologist at the time. Both concluded she was competent to make this decision and this was noted in her medical records. The patient discussed her wishes extensively with her family. After witnessing her mother and two of her brothers suffer from dementia in a nursing home, she decided she never wanted a similar fate for herself. The AED was reconfirmed at the GP visits over the following years (during regular three monthly checks). The patient updated the dementia clause in 2015; the content was nearly the same as the previous version:

I would like to use the legal right to be given voluntary euthanasia, when I think the time is right. I do not want to be placed in a nursing home for elderly people with dementia. I want to say goodbye to my loved ones in a timely and dignified manner. My mother in her time had been in a nursing home for 12 years before she died, so I have close experience of it. I know what I am talking about. I definitely do not want to experience this, it has traumatised me severely and really saddened the whole family. Trusting, that when the quality of my life is so low, that at my request euthanasia will be performed.

Decline set in during 2015. The patient was judged incompetent with respect to her euthanasia request by her GP in January 2016. Shortly thereafter she was admitted to a nursing home.

The nursing home physician was told by the patient’s husband that she had an AED. She investigated whether euthanasia on the basis of this AED was possible. She read the medical records, repeatedly spoke with and observed the patient. She discussed the case with the (former) GP, the family, the treatment team of the nursing home, the patient’s psychologist and an expert from the Expertise Centrum Euthanasie.^5^

She concluded that the patient was experiencing a complete breakdown of her person and was suffering unbearably:

The patient was agitated, restless, stressed, fearful, sad, angry and panicky. She cried a lot, repeatedly said she loathed it and that it destroyed her, and stated up to twenty times a day that she wished to die. Her day-night rhythm was disturbed and she roamed the corridors day and night. She hammered on doors and windows until her hands hurt. (…) This [her behaviour] led to physical conflicts with other residents. She also underwent physical personal loss of dignity, because of her great dependence and incontinence. (From the verdict, translation by authors)^6^

Medication failed to reduce this suffering.

As required the physician further consulted two independent physicians whether granting this euthanasia request (based on the AED and her current state) would meet the due care criteria, both agreed this was the case. In April 2016 she performed the euthanasia. In order to avoid confusion and (apparent) resistance the physician sedated the patient before the euthanasia, mixing the sedative in the patient’s morning coffee. These steps were discussed in advance with the family. The actual euthanasia was not discussed with the patient at that time, and the patient did not know she was about to die. During the performance of the euthanasia, the patient did respond physically to the administration of the medication, by sitting up despite the sedative. The patient was restrained by her family during the further performance of the euthanasia.

## Earlier verdicts on this case

### Regional Review Committees for Termination of Life on Request and Assisted Suicide

The regional committees judged the case as not meeting the due care criteria. In particular, they considered the due care criteria of the voluntary and well-considered request and the professional and careful performance of the euthanasia were not met.

The RTE thought the clarity of the wording of the AED was lacking. They considered it multi-interpretable, as either the wish to have euthanasia at the time of admittance to a nursing home or at a time of the patient’s own choosing before admittance. Considering this ambiguity and the fact that the patient was now incompetent with respect to this decision, the RTE considered it prudent to err on the side of caution and not perform euthanasia in such a case. As there was no actual (and oral) euthanasia request at the time of the euthanasia, and the AED was not sufficiently clear, the RTE concluded there was no (unequivocal) voluntary and well-considered request.^7^

Second, the RTE raised concerns about the manner in which the euthanasia was performed, particularly the use of a sedative to ensure the patient would not resist the performance of the euthanasia, and that it is was slipped in the coffee. Moreover, when the patient reacted to the administration of the medication, the RTE judged the patient might have been resisting euthanasia. Any doubt should lead to a reconsideration of the performance of the euthanasia. Resistance to the procedure (involving needles, and so on) should, according to the RTE, be taken seriously, even in a patient incompetent to understand euthanasia and the goal of the procedure. Thus, the RTE judged the criterion of professional and careful administration as not met.^7^

Taken together the RTE thus judged the case as not meeting the requirements of due care and it was duly forwarded to both the Health Care Inspectorate and Criminal Prosecution for investigation. Both did so and the disciplinary court came to a final verdict (after appeal) before the case was brought to the criminal court.

### Disciplinary court

The medical disciplinary court tested the euthanasia against the guidelines for professional conduct, which are based on the legal requirements, but not exactly the same. These guidelines, both those drafted by the Royal Dutch Medical Association (RDMA) and the Euthanasia Code issued by the RTE, state that there should be an attempt to discuss an actual wish to die at the time of the euthanasia; this is not found in the Act itself.^2 8^ The disciplinary court follows the guidelines of the RDMA as they stand, even though there is room for discussion of this particular requirement. It is difficult to see whether an attempt to discuss the euthanasia could have been productive. The physician in this case said she made the conscious decision not to discuss the euthanasia as she believed the patient would not understand and only be unduly stressed.

The disciplinary court also concluded that the AED was ambiguous and that considering the gravity of the act of euthanasia, there is no room for interpretation.^9^ Thus, an ambiguous AED cannot be sufficient to decide on the performance of euthanasia, and therefore the physician did not meet the criterion of ‘voluntary and well-considered request’. Interestingly, the disciplinary court did mention that a consistent and actual death wish of the patient at the time of the euthanasia could have allowed her to conclude that there was a voluntary and well-considered request (even though the patient was incompetent). Moreover, the disciplinary court held that the physician should have attempted to communicate the intended euthanasia with her, and not slipped a sedative in her coffee, even though the court accepts that the patient might not have understood this and it may have caused distress in the patient. The court considered a warning (the lightest possible measure) appropriate, because the physician had done extensive research and consultation before she came to her conclusions and she had been open and transparent about her actions and the reasons she had for them.^9^

## Judgement of the criminal court

In 2019 the case was brought before the criminal court; the public prosecutor focused on the voluntary and well-considered request requirement. The validity of the advance directive was questioned, and the fact that the physician had not sought the patient’s consent for the euthanasia. The prosecutor referred to the Position Paper drafted by the RDMA, which states that professional norm requires communication with the patient until the last moment.^8^ The prosecutor therefore stated that the physician should have tried to discuss the euthanasia with the patient, even though she was deeply demented and incompetent. In the criminal court the physician was acquitted of all charges and the court held that the physician did meet all the due care criteria. Here the considerations of the court are described in more detail.^6^ The prosecution has decided to take the case to the High Court for a Cassation in the interest of the Law; the physician will remain acquitted.

### Wording of the AED

The judgement was that the AED was clear enough, especially in the context of the patient’s ongoing conversation about euthanasia with her GP and a discussion of her euthanasia wish and the AED with her treating gerontologist, at a time both the GP and the gerontologist judged the patient was still competent. The concerns raised on the apparent ambiguity, namely the implied wish to choose her own time of death in the dementia clause, while at the same time definitely stating that she did not want to be placed in a nursing home, were not shared by the court.

The court judged that her desire for euthanasia once she needed to be admitted to a nursing home was completely clear *in context*, even though she might have preferred to be aware enough to have chosen her own time before. The context concerned her repeated discussion and confirmation of the euthanasia wish with her GP, her stated wish to the gerontologist and her repeated conversations with her husband and daughter.

The court moreover judged that oral confirmation of the actual wish to have euthanasia is impossible in a patient with advanced dementia who can neither understand her own disease, nor death or euthanasia. The court notes that this requirement for oral confirmation is not in the law, legal history or case law, and is therefore moot. Whether the RDMA will revise their guidelines to reflect this remains to be seen. Given that a deeply demented patient cannot understand euthanasia and what is practically happening, it can be best to premedicate (sedate) the patient before the euthanasia procedure to avoid undue stress to the patient.^6^

## Consequences of the judgement

### The AED

This verdict means that patients with advanced dementia in the Netherlands can still be granted euthanasia on the basis of an AED, if the AED was written while the patient was still competent. This AED does not need to have the (legal) clarity sought by the RTE or the disciplinary court. Instead the AED can be read and interpreted in the context of the conversations with the patient noted in the medical records and the testimony of the family. This means that not everyone needs to consult a lawyer to write an AED. However on-going conversations and affirmations about the content of the AED with their physician are needed because further interpretation of the AED is usually necessary. In addition, a judgement of competence is necessary to decide whether the AED is in principle valid. The verdict now legally allows the interpretation of an AED based on conversations and clarifications made to their physician and others. Thus, the verdict helps overcome one of the intrinsic difficulties of drafting an AED, namely to be clear enough for the performance of euthanasia, and not too restrictive.

### Whose interests or which interests?

The judgement thus weighs in on one of the big discussions about advance directives in general, and AEDs in particular. There are those who argue that euthanasia is indefensible after an AED because the trajectory should be a collaborative enterprise between the physician and the patient.^10^ Some even claim an AED should only be used as a tool in shared decision-making,^11^ which seems counterintuitive.

If one however accepts the idea of an AED, the question remains whose interests are at stake and should be followed. This is discussed extensively in the literature and often described as the ‘then-self versus now-self’’ problem.^12 13^ The core question here is whether one should follow the wishes of the then-self as put down in the AED or follow the apparent interests of the person with dementia. Some claim the now-self is a new person, based on the huge changes in psychological identity, and that this new and vulnerable person should be protected even from his former wishes.^14^ Sometimes this discontinuity in psychological identity is taken as a sign of adaptation to the disease,^13 14^ although this is also much contested. Developing new values and interests in these cases is unlikely as a result of developing dementia. It has been argued that dementia does not cause a change in a person’s preferences, values or internal standards, but rather a loss of all of this as a result of cognitive decline.^15^

Alternatively, it can be argued that the opposition between the now-self versus the then-self is a false dichotomy. If one accepts the person with dementia as the same person as before, because the person is the same character in one ongoing life story with one narrative identity, it is not implausible to take account of the wishes expressed before. Den Hartogh views the person with dementia as ongoing and continuous with respect to the story of her life. He argues the most important reason to write an AED for dementia is the wish to not end one’s life story on such a completely different track, that is, with severe dementia in a nursing home.^16^ The question then becomes which interests take precedence. If one emphasises the importance of critical interests—as coded in the AED—versus experiential interests—as expressed by or observed from the patient, the earlier critical interests can take precedence.^17^

The verdict affirms that for the law the now-self and the then-self are the same person, otherwise AEDs or any other advance directive could never be valid. Moreover, using the context to inform the interpretation of the AED appears in line with an account based on narrative identity. The judgement however does not mean that apparently happy, persons with advanced dementia can be receiving euthanasia in the Netherlands. In order for a physician to perform euthanasia, he or she must be convinced the patient in question is suffering unbearably and hopelessly at the time of the euthanasia. In Dutch law both earlier and current interests need to be in line to grant euthanasia on the basis of an AED. Despite ample discussion on the difficulties of assessing suffering in a patient with severe dementia, no one had doubts about the suffering in this case

### Following an AED in the absence of suffering

Although Dutch law does not allow it, we would argue that even in the case of a person with advanced dementia, who is not noticeably suffering, an AED (morally) should be followed. We believe that the (apparent) experiential interests of a person should not over-rule the existential interests laid down in an AED. Because it is unclear exactly what the interests of the person with advanced dementia are, we can only assume on the basis of observation. It is impossible to exclude that it might still be in the person’s interest to no longer exist in the state of advanced dementia considering the narrative unity of his life despite the apparent interests or absence of apparent suffering.

Moreover, the person himself is no longer able to reflect on his interests and weigh them himself. This weighing should have happened at an earlier stage when the person still had the mental faculties necessary. The presence of an AED (and its context in conversations) is a clear sign that this person considered the interest to avoid a particular state, namely advanced dementia, as his most important. During the drawing up of an AED the person would have (or should have) considered that he might be an apparently happy person with advanced dementia, and still considered this not to be the end of his narrative that he wanted. In fact many people with AEDs consider the idea that they will be happily watching children’s television or smearing shit on the wall as a nightmare and—for them—against their dignity. Thus, if we consider the person as continuous, we can grant this person, when he was still competent, the responsibility to guide us, as to which interests should take precedence once he is incompetent.

Any change of the law to accommodate our view would however probably lead to protests from Dutch physicians, a number of whom have argued that they find it morally repulsive to help die someone who is unaware of this. In fact, before this case went to court, 220 physicians placed a large advertisement in major Dutch newspapers, stating that performing euthanasia for anyone unaware is something they will never do.^18^

### Being granted euthanasia becomes more difficult

The wider impact of this verdict may ultimately lie in the fact the physician was prosecuted in a criminal court. Even though she was acquitted, the fear of being prosecuted deters Dutch physicians from granting euthanasia requests, even for other, far less controversial cases. The Dutch Centre for Euthanasia Expertise (before: ‘End-of-life clinic’) observed a stark increase in the number of requests they received since the prosecution was announced and a shift towards more relatively straightforward cases being referred to them, for instance concerning patients with terminal cancer.^5^

## Conclusion

The end result of this judgement is that the AED is still standing for patients with advanced dementia in the Netherlands, and thus it is theoretically possible to be granted euthanasia at an advanced stage of dementia. However, being granted euthanasia on the basis of an AED with advanced dementia is not easy and there are a number of reasons for this.

Besides the need for an AED, preferably supported by repeated conversations with physicians and family while still competent, there is the requirement of unbearable suffering. This means that people with crystal clear AEDs cannot be granted euthanasia if they are apparently not suffering in the advanced stages of dementia.

Moreover, physicians experience a serious moral boundary with euthanasia on the basis of an AED for patients with advanced dementia. Many requests for euthanasia are the beginning of a shared process, in which physician and patient together explore the suffering, the (lack of) possibilities to alleviate that suffering and together reach the conclusion that there is no other means to reduce the suffering than euthanasia. In the case of an AED the physician partly has to go through this process alone, once the patient becomes incompetent and therefore no longer a real party to the process. The importance of communication and togetherness is often stated by physicians and should be taken seriously.^8 10 11^ It does mean that not many physicians are willing to perform euthanasia on a patient with advanced dementia. The few who are willing in principle might be deterred by this prosecution. So despite the outcome of the trial, it has become harder to be granted euthanasia in these difficult cases.
